# Pathogenesis Study of Glioma: From Glioma Stem Cells, Genomic Tags, to Rodent Models

**DOI:** 10.3390/brainsci13010030

**Published:** 2022-12-23

**Authors:** Hailiang Tang, Xi Li, Rong Xie

**Affiliations:** 1 Department of Neurosurgery, Huashan Hospital, Shanghai Medical College, Fudan University, No. 12 Wulumuqi Middle Road, Shanghai 200040, China; 2 National Center for Neurological Disorders, Shanghai 200040, China; 3 Shanghai Key laboratory of Brain Function and Restoration and Neural Regeneration, Shanghai 200040, China; 4 Neurosugical Institute, Fudan University, Shanghai 200040, China; 5 Shanghai Clinical Medical Center of Neurosurgery, Shanghai 200092, China; 6 Shanghai Pulmonary Hospital, Tongji University, Shanghai 116085, China

Glioma remains the toughest brain tumor among all primary central nervous system (CNS) tumors. The complexity of its pathogenesis makes it difficult to achieve radical cure, especially in the case of glioblastoma multiforme (GBM, WHO grade IV), the most aggressive subtype of glioma [1]. Recently, the existence of glioma stem cells (GSCs) in GBM has been demonstrated, which exhibit the properties of neural stem cells (NSCs) and are responsible for chemo/radiotherapy resistance and tumor recurrence [2,3,4], making them potential therapeutic targets against GBM [5,6].

In this Special Issue, we collected 10 articles discussing the genomic alterations of glioma, its immune microenvironment, and multi-therapy for glioma. *Wu.* et al. screened necroptosis (NCPS)-related genes (CTSD, AP1S1, YWHAG, and IER3) to construct a prognostic model for GBM, paving the way for the use of new targets for the diagnosis and treatment of glioma. *Fang* et al. proved that the transient receptor potential (TRP) family genes are promising immunotherapeutic targets and potential clinical biomarkers for glioma. Besides genetic mutations, increasing evidence shows that the tumor microenvironment is also important for glioma development and resistance to therapy [7]. Within the GBM microenvironment, *Alice Giotta Lucifero* et al. found that the phosphatase and tensin homolog (PTEN)-related immune landscape mainly consists of T_reg_ and M2 macrophages, which repress the antitumor immune activation and are responsible for triggering glioma cell growth and invasion. *Li* et al. demonstrated that the tyrosine phosphatase receptor type N (PTPRN) could be an independent prognostic factor and correlates with tumor immune infiltration in low-grade glioma.

In the past decade, newly emerging therapeutic strategies such as tumor-treating fields (TTF) have been introduced in GBM patients [8]. *Yu* et al. suggested that irreversible electroporation possibly mediates glioma apoptosis via the upregulation of transcription factor AP-1 and Bim (Bcl2l11) expression. Programmed death protein 1 (PD-1) and programmed death-ligand 1 (PD-L1) play critical roles in tumor immune escape, and several immunotherapies, including PD-1/PD-L1 checkpoint inhibitors and chimeric antigen receptor-T cells (CAR-T), have already been applied in glioma therapy. *Yu* et al. explore the PD-1/PD-L1 protein expression in recurrent glioma and its paired primary tumor and reveal a tendency of increased PD-1/PD-L1 in recurrent glioma. However, immunotherapies proved therapy failures, which strongly indicates that beyond the T cell-based adaptive immunity, innate immunity might also be the key to regulating anti-tumor immunity in the glioma microenvironment. *Qi* et al. provided a cellular response to the interleukin-4 (IL-4)-related gene signature as an excellent immune biomarker of gliomas, which may be beneficial to develop novel immunotherapies for glioma.

Rodent models of glioma are still indispensable for understanding the basic principles of glioma development and tumor invasion [9]. Rodent models, including xenograft (Figure 1) and genetically engineered models, are used to study glioma development, reveal tumor progression, and test novel therapy strategies [10,11]. *Cintia Carla da Hora* et al. proposed patient-derived xenografts (PDX) or patient-derived GSC models in glioma research, which provide the possibility of studying glioma growth, treatment response, and survival outcome. In addition, *Hannes Becker* et al. conducted multilayered profiling of a platelet-derived growth factor B (PDGFB)-driven glioma mouse model and discovered radiological, histological, and metabolic features that are comparable to human high-grade glioma.

## Figures and Tables

**Figure 1:**
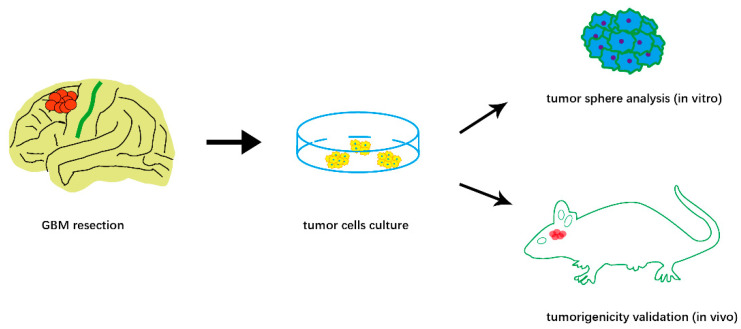
Schematic illustration of patient-derived xenografts (PDX) to study GBM biology.

## Data Availability

Not applicable.
